# Doppler myocardial performance index combined with plasma B-type natriuretic peptide levels as a marker of cardiac function in patients with decompensated cirrhosis

**DOI:** 10.1097/MD.0000000000013302

**Published:** 2018-11-30

**Authors:** Li-Kun Wang, Xiao-Fei An, Xue-Liang Wu, Su-Mei Zhang, Rui-Min Yang, Chao Han, Jie-Lin Yang, Yi-Cheng Wang

**Affiliations:** a Department of Ultrasound, The First Affiliated Hospital of Hebei North University, Zhangjiakou; b Department of Intensive Care Unit, Daqing Oilfield General Hospital, Daqing; c Department of General Surgery, The First Affiliated Hospital of Hebei North University; d Department of Ultrasound, The People's Hospital of XuanHua District of Zhangjiakou City, Hebei Province; e Department of Gastroenterology, The First Affiliated Hospital of Hebei North University, Zhangjiakou, PR China.

**Keywords:** cardiac function, cirrhosis, decompensated stage, Doppler myocardial performance index, plasma B-type natriuretic peptide levels

## Abstract

**Background:**

In chronic liver diseases, cirrhosis ranks as the 14th highest death cause worldwide, developing into decompensated cirrhosis. A potential and feasible technique in assessing cardiac function is urgent. This study explores if the Doppler myocardial performance (Tei) index combined with the plasma B-type natriuretic peptide (BNP) levels can assess cardiac function in patients with decompensated cirrhosis.

**Methods:**

A total of 140 individuals were selected in the study and were classified into 3 groups: control group (n = 40, healthy individuals), compensated cirrhosis group (n = 50), and decompensated cirrhosis group (n = 50). Plasma BNP levels, alanine aminotransferase (ALT), aspartate aminotransferase (AST), total bilirubin (TBIL), and albumin (ALB) were identified by an enzyme-linked immunosorbent assay (ELISA). The correlation of Tei index between left ventricle (LV) and right ventricle (RV) as well as plasma BNP levels with cardiac function was assessed using a Pearson test analysis. All patients were subjected to this experiment for 1 year to analyze the relationship between Tei index and plasma BNP levels in prognosis of decompensated cirrhosis patients.

**Results:**

Patients with decompensated cirrhosis showed significantly elevated levels of ALT, AST, and TBIL level in contrary to a reduced ALB level. Cirrhosis patients also showed a significantly reduced ejection fraction (ET) index, but an increase in isovolumetric contraction time (ICT), isovolumetric relaxation time (IRT), Tei index, and plasma BNP levels in comparison to healthy individuals. ICT, IRT, Tei index, and plasma BNP levels were elevated in decompensated cirrhotic patients as opposed to compensated cirrhotic patients. These results indicate a positive correlation of both Tei index and plasma BNP levels with cirrhosis and its progression. Tei index and plasma BNP levels are positively associated with Child–Pugh classification and negatively correlated with both cardiac function and prognosis in patients suffering from decompensated cirrhosis.

**Conclusion:**

The study provided evidence supporting the correlation of Tei index and plasma BNP levels in decompensated cirrhotic patients with cardiac function, highlighting a potential value for evaluation.

## 1 Introduction

Cirrhosis is caused by the long clinical progression of chronic liver diseases and is expressed by the conversion of normal liver architecture into structurally abnormal nodules and tissue fibrosis.^[1]^ Cirrhosis ranks as the 14th common cause of death worldwide, with 1-year mortality rates ranging between 1% and 57%, depending on the stage.^[2]^ Patients with cirrhosis are immunocompromised and susceptible to infection because of the multimodal defects involved with the innate immune system.^[3]^ Moreover, cirrhosis is accompanied with heart failure, which is manifested due to increased cardiac output, as well as a blunted systo-diastolic response.^[4]^ Cirrhotic cardiomyopathy, a clinical syndrome in cirrhosis patients with abnormal heart structure and function, is considered a serious complication.^[5]^ Cirrhosis usually develops into decompensated cirrhosis which is characterized by hepatic encephalopathy, variceal bleeding, and ascites.^[6]^ Decompensated cirrhosis is likely caused by portal hypertension and/or liver insufficiency.^[7]^ Fortunately, developing studies focusing on the evaluation of cardiac function in cirrhotic patients have found that myocardial contractility is indicated in predicting the prognosis of patients with decompensated cirrhosis.^[8,9]^ Furthermore, a potential and feasible technique to assess cardiac function might be of useful in patients with decompensated cirrhosis.

Doppler myocardial performance index (MPI/Tei index) is regarded as a useful predictor of both systolic and diastolic function.^[10]^ The Tei index consists of the isovolumetric contraction time (ICT) and isovolumetric relaxation time (IRT) divided by the ventricular ejection time (VET) in each ventricle (ICT + IRT)/VET.^[11]^ Karasek et al^[12]^ have reported that the normal Tei index should be <0.4, and any increase exhibited is associated with depraved ventricular function. The degree of cardiac failure can be measured not only by assessing cardiac function using the Tei index, but also by detecting the B-type natriuretic peptide (BNP) levels.^[13]^ BNP is cardiac hormone secreted by cardiomyocytes in the heart ventricles in response to increased cardiac wall stress caused by increased ventricular blood volume.^[14]^ It has natriuretic, diuretic, and vasodilator actions regulating volume homeostasis and blood pressure.^[15]^ Furthermore, BNP levels are accompanied with degree of left ventricle (LV) hypertrophy, LV outflow gradient, LV diastolic dysfunction, and LV systolic impairment.^[16]^ BNP levels are relatively high in congestive states such as heart and renal failure as well as chronic liver disease, specifically cirrhosis.^[17]^ Although BNP was applied in the evaluation of cardiac function in cirrhosis,^[17]^ the performance of Tei index combined with plasma BNP levels in evaluation of cardiac function remains unknown in patients with decompensated cirrhosis. Therefore, the present study was performed to investigate the association between Tei index and plasma BNP levels with cardiac function in decompensated cirrhosis patients and the evaluation value of both the plasma BNP levels and Tei index.

## 2 Materials and methods

### 2.1 Study subjects

A total of 100 subjects who were diagnosed with cirrhosis and hospitalized at the Gastroenterology Department of The First Affiliated Hospital of Hebei North University between June 2014 and June 2016 were enrolled in the current study. According to the Child–Pugh classification in cirrhosis^[18]^ and clinical symptoms, the patients were allocated into both compensated cirrhosis (n = 50) and decompensated cirrhosis groups (n = 50). The inclusion criteria went as follows: patients aged between 30 and 60 years old; diagnosis of compensated cirrhosis: Child–Pugh A, mild fatigue, loss of appetite or abdominal distension, no obvious liver failure, decreased albumin levels (ALB, still ≥ 35 g/L), bilirubin < 35 μmol/L, prothrombin activity more than 60%, slightly increased serum alanine aminotransferase (ALT), aspartate aminotransferase (AST), r-aminopeptidase, mild portal hypertension, and no ascites, hepatic encephalopathy, or upper gastrointestinal (UGI) bleeding; diagnosis of decompensated cirrhosis: Child–Pugh B and C, overt hepatic dysfunction, and decompensation, such as ALB < 35 g/L, A/G < 1.0, manifest jaundice, bilirubin > 35 μmol/L, increased ALT and AST, prothrombin activity < 60%, and ascites, hepatic encephalopathy, varices or rupture bleeding of esophageal or gastric fundus caused by portal hypertension. In contrary, the exclusion criteria went as follows: patients with hemorrhagic cerebrovascular disease; patients with primary heart and lung complications, severe thyroid conditions, diabetes, and kidney disease; patients with mental disorder or dementia. Additionally, 40 healthy people who received physical examination in The First Affiliated Hospital of Hebei North University during the same period were selected as part of the control group. The present study was approved and supervised by the ethic committee of The First Affiliated Hospital of Hebei North University and written informed consent was obtained from patients and/or family members.

### 2.2 Cardiac function evaluation and ascites ultrasonic examination

An Aloka 5500 ultrasonic diagnostic apparatus (Hitachi Aloka Medical, Tokyo, Japan) with probe frequency ranging between 2 and 5 MHz was applied for a routine cardiac ultrasonography which detected 4 cardiac indexes: stroke volume (SV), ejection fraction (EF), the E/A ratio of peak early diastolic flow velocity (E velocity), and the peak flow velocity of atrial contraction (A velocity). During examination, the patients were instructed to lie in the left lateral position to breathe comfortably. When the patients lay in supine position, the maximum depth of pelvic ascites was measured under two-dimensional ultrasound modalities. The depth being < 3 cm was a small amount of ascites, 3 to 5 cm was medium ascites, and ≥ 5 cm was massive ascites.

### 2.3 Tei index

After conducting a routine cardiac ultrasonography, the patients were treated with conventional apical four-chamber view, five-chamber view, and arterial short-axis view. The sampling volumes were placed in the mitral, aortic, 3 cusp, and pulmonary valve orifices. The instrument filler and contrast were adjusted to visibly display the blood flow spectrum at the beginning, half entrance, and the stop point, along with the QRS Wave Group's Time Limit of Electrocardiograph (ECG). A total of 3 to 5 cardiac cycles were recorded by blood flow spectrum of each flap, and the Tei index was analyzed with an image replay in the E-DMS system. The blood flow spectrums of both mitral and aortic valves were examined by Tei index, with the ICT, IRT, ET, and Tei index (Tei index = [ICT + IRT]/ET) of the left ventricular being obtained. The blood flow spectrums of both the heart and pulmonary valve were examined to evaluate the Tei index and parameters of the right ventricle. No ICT and IRT were observed in patients with mitral regurgitation, which made it possible to replace the interval of the closing mitral valve to opening aortic valve and the interval of closing aortic valve to the opening mitral valve.

### 2.4 Measurement of plasma BNP levels and liver function indexes

A total of 5 mL of fasting blood sample was extracted from all patients on either the day of admission or early morning of the next day, however, patients in the control group had it extracted during their physical examination. In order to obtain the blood sample, a peripheral venipuncture was used to procure the blood from the antecubital fossa. An enzyme-linked immunosorbent assay (ELISA) was applied to determine plasma BNP levels with B type N-terminal pro-brain natriuretic peptide assay kit (Nanjing Jiancheng Bioengineering Institute, Nanjing, China). Finally, a biochemical immunity integrative system was used to detect liver function indexes, including ALT, AST, total bilirubin (TBIL), and serum albumin (ALB).

### 2.5 Follow-up

The patients had follow-ups done that included telephone calls, outpatient visits, or medical records. Most patients had follow-ups for 3 to 12 months, which stopped in June 2017. However, there were 5 censored cases, meaning these patients were lost to follow-up, and in return dropping the follow-up rate to 90% (45 out of 50 total cases). The prognosis of patients with decompensated cirrhosis was evaluated at the end.

### 2.6 Statistical analysis

Statistical Package for the Social Sciences (SPSS) 17.0 software (SPSS Inc., Chicago, IL) was used for interactive, or batched statistical data analysis. The measurement data were presented by the mean ± standard deviation. Descriptive statistics were used for the distribution of the data. A *t*-test was applied for to compare 2 groups whose measurement data obeyed normal distribution and a one-way analysis of variance (ANOVA) was useful in differentiating among multiple groups, which were further tested with a Tukey post-hoc test. Enumeration data were expressed as a percentage and both chi-square test and rank sum test were used for comparison. A Pearson correlation analysis was used to investigate the correlation of Tei index and plasma BNP levels. A value of *P* < .05 was considered to have a statistically significant difference.

## 3 Results

### 3.1 Plasma TBIL level is increased, and plasma ALB level is decreased in patients with compensated cirrhosis

Among the 40 individuals involved in the control group (26 males and 14 females), the average age was 47.48 ± 4.62 years old. The 50 patients with compensated cirrhosis (38 males and 12 females) had an average age of 46.70 ± 3.36 years and lastly, the 50 patients with decompensated cirrhosis (35 males and 15 females) had an average age of 48.44 ± 4.13 years. There were no significant differences noticed in age, sex composition, and BMI index among the 3 groups (all *P* > .05). In comparison with the control group, the decompensated cirrhosis group showed significantly elevated levels of ALT, AST, and TBIL, but a reduced ALB level (all *P < *.05). In addition, the TBIL level was significantly higher, but ALB level was found to be lower in the compensated cirrhosis group than in the control group (all *P* < .05). Levels of ALT and AST were both slightly elevated in compensated patients in comparison to the control group (all *P > *.05) (Table 1).

**Table 1:**
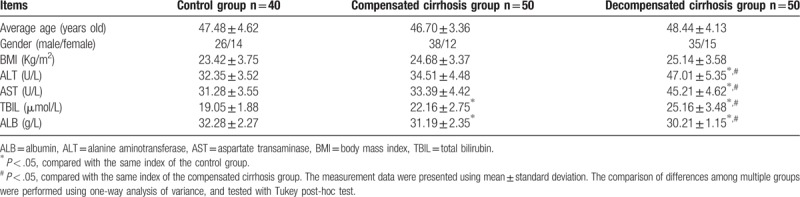
Clinical characteristics and liver function indexes among healthy individuals, compensated cirrhotic, and decompensated cirrhotic patients.

### 3.2 Tei index and plasma BNP levels are associated with cirrhosis and its progression

There were no obvious differences in the ICT, IRT, ET, and Tei index between both the left (LV) and right ventricular (RV) of each group (all *P > *.05). Cirrhosis patients displayed significantly reduced ET in both the LV and RV (all *P < *.05) as opposed to the control group. The ET index of both LV and RV was drastically lower in the decompensated cirrhosis group in comparison to the compensated cirrhosis group (all *P < *.05). Additionally, cirrhosis patients had significantly increased ICT, IRT, and Tei index of both LV and RV (all *P < *.05) opposed to the control group. The ICT, IRT, and Tei index of LV and RV were significantly increased in the decompensated cirrhosis group in comparison to the compensated cirrhosis group (all *P < *.05). When making the differentiation with the control group, cirrhosis patients had significantly increased plasma BNP levels (all *P < *.05), and the ensuing plasma BNP levels were higher in the decompensated cirrhosis group than those in the compensated cirrhosis group (all *P < *.05). These results indicate that the Tei index and plasma BNP levels were associated with cirrhosis and its progression (Table 2).

**Table 2:**
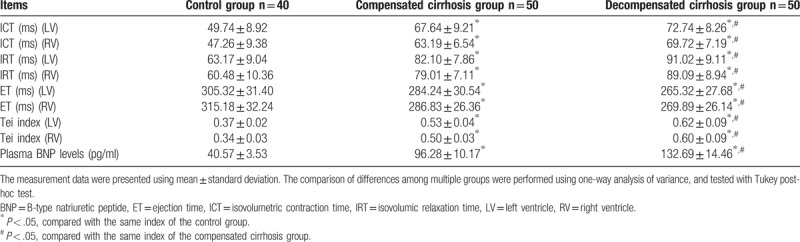
Tei indexes and plasma BNP levels in LV and RV of healthy individuals, compensated cirrhotic, and decompensated cirrhotic patients.

### 3.3 The most serious cardiac damage occurs in patients with decompensated cirrhosis

In comparison with the control group, cirrhosis patients elucidated signs of obvious impairment of cardiac function. The impairment of cardiac function in the patients with decompensated cirrhosis was significantly higher than in patients with compensatory cirrhosis. The decompensated cirrhosis group also had significantly decreased SV, EF, and E/A values (all *P < *.05) (Table 3).

**Table 3:**
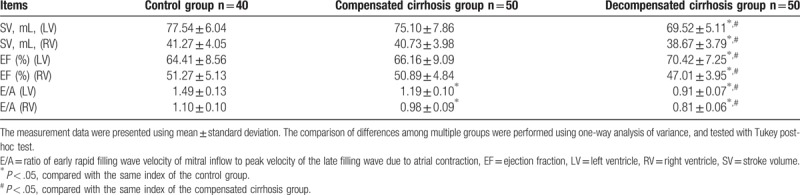
Cardiac function indexes in LV and RV of the healthy individuals, compensated cirrhotic, and decompensated cirrhotic patients.

### 3.4 Tei index is positively correlated with plasma BNP levels in patients with decompensated cirrhosis

**Figure 1:**
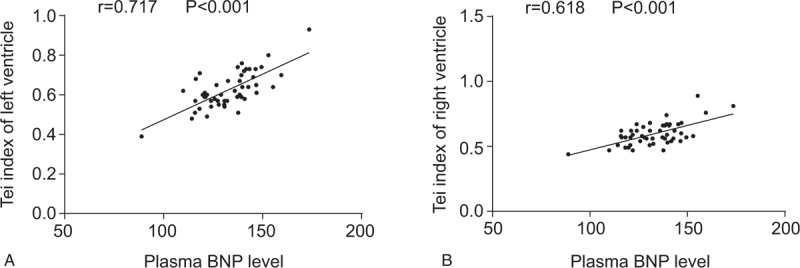
Pearson correlation analysis of Tei index of LV and RV with plasma BNP level in patients with decompensated cirrhosis. (A) Correlation of Tei index of LV with plasma BNP level in patients with decompensated cirrhosis. (B) Correlation of Tei index of RV with plasma BNP level in patients with decompensated cirrhosis. Pearson correlation analysis was used to analyze their correlations. BNP = B-type natriuretic peptide, LV = left ventricle, RV = right ventricle.

A Pearson correlation analysis was conducted to determine the correlation between Tei index of LV and RV with the plasma BNP levels in patients with decompensated cirrhosis. The results (Fig. 1) revealed that the Tei index of LV and RV were both positively associated with plasma BNP levels in patients with decompensated cirrhosis (*r* = 0.717, *P < *.001; *r* = 0.618, *P < *.001).

### 3.5 Tei index and plasma BNP level are higher in patients classified with Child–Pugh C compared with B

According to the Child–Pugh classification, patients diagnosed with decompensated cirrhosis were assigned into Child–Pugh B and Child–Pugh C. The Tei index of LV and RV was elevated in the decompensated cirrhosis patients with Child–Pugh C than in the decompensated cirrhosis patients with Child–Pugh B (all *P < *.05). The plasma BNP levels were also higher in the decompensated cirrhosis patients with Child–Pugh C than in the decompensated cirrhosis patients with Child–Pugh B (all *P < *.05) (Table 4).

**Table 4:**
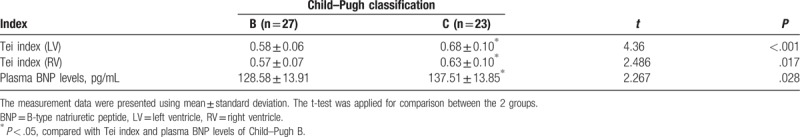
Tei index in LV and RV and plasma BNP levels increased in patients with decompensated cirrhosis classified with Child–Pugh C.

### 3.6 Tei index and plasma BNP levels are negatively correlated with cardiac function in patients with decompensated cirrhosis

**Figure 2:**
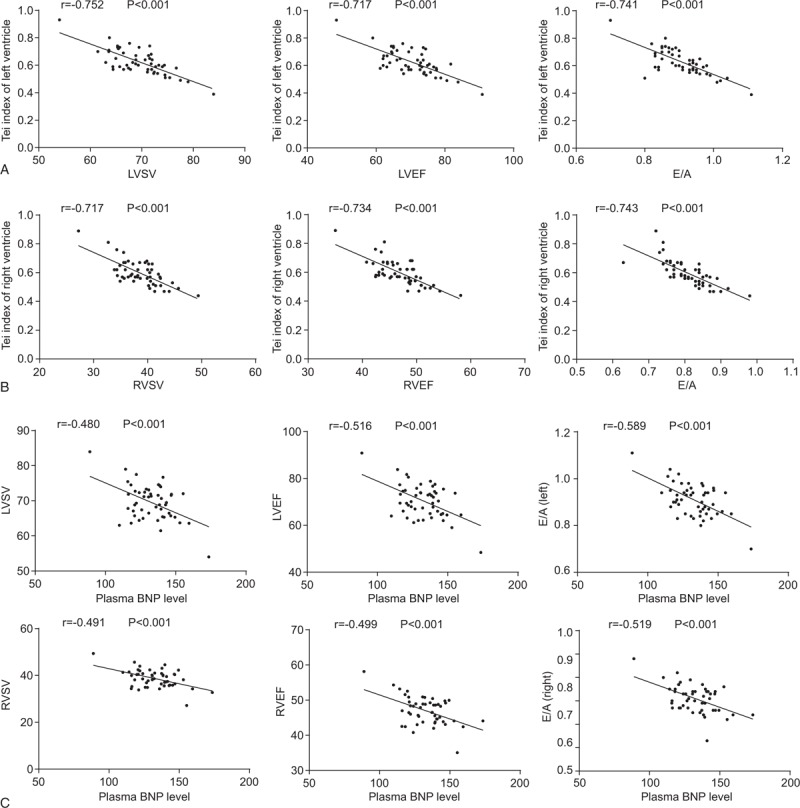
Pearson correlation analysis of Tei index of LV and RV and plasma BNP level with cardiac function in patients with decompensated cirrhosis. (A) Correlation of Tei index of LV with cardiac function in patients with decompensated cirrhosis. (B) Correlation of Tei index of RV with cardiac function in patients with decompensated cirrhosis. (C) Correlation of plasma BNP level with cardiac function in patients with decompensated cirrhosis. Pearson correlation analysis was used to analyze their correlations. BNP = B-type natriuretic peptide, EF = ejection fraction, LV = left ventricle, RV = right ventricle, SV = stroke volume.

As mentioned, a Pearson correlation analysis was employed to assess correlations between Tei index and plasma BNP levels with cardiac function in patients involved with decompensated cirrhosis. In the patients suffering from decompensated cirrhosis, Tei index of LV showed a negative correlation with LVSV, E/A, and LVEF, while Tei index of RV was negatively correlated with RVSV, E/A, and RVEF (all *P < *.05). The plasma BNP levels were also negatively correlated with LVSV, E/A, LVEF, RVSV, E/A, and RVEF (all *P < *.05) (Fig. 2). Thus, due to high Tei index in LV and RV and high plasma BNP level, there might be sufficient evidence to suggest correlation with damaged cardiac function in patients with decompensated cirrhosis.

### 3.7 Tei index and plasma BNP levels are higher in dead patients with decompensated cirrhosis compared with survivors

The follow-up rate of the 50 patients with decompensated cirrhosis was 90% (45 cases), and the 1-year survival rate of the active 45 patients with decompensated cirrhosis was listed at 86.7% (approximately 39 cases). The dead patients that had suffered from decompensated cirrhosis had an increased Tei index of LV and RV in comparison to patients who survived. Additionally, dead patients with decompensated cirrhosis had increased plasma BNP levels in comparison with that of surviving patients (all *P < *.05) (Table 5). Therefore, both Tei index and plasma BNP levels were markedly increased in patients with decompensated cirrhosis with a poor prognosis.

**Table 5:**
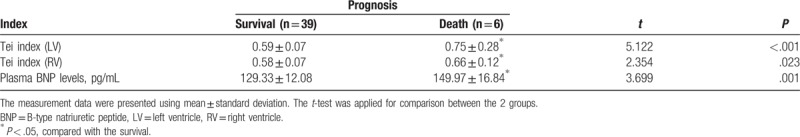
Tei index in LV and RV and plasma BNP levels were elevated in dead patients with decompensated cirrhosis than survivors.

## 4 Discussion

It is well known that cirrhosis has an increased risk of infection and poor prognosis.^[19]^ Tei index was applied to detect the systolic dysfunction of both ventricles in cirrhosis patients receiving liver transplantation.^[20]^ In addition, BNP has been negatively associated with the cardiac function in cirrhosis patients.^[21]^ The present study was conducted in order to evaluate the cardiac function involved in patients with decompensated cirrhosis by using a Tei index combined with the present plasma BNP levels. The findings obtained provided enough evidence to support the negative correlation between the Tei index and plasma BNP levels in decompensated cirrhosis patients with cardiac function, progression, and prognosis.

The study implicated that patients with decompensated cirrhosis showed elevated levels of ALT, AST, and TBIL, but a reduction in ALB levels in comparison to healthy individuals. Furthermore, patients with compensated cirrhosis had a significantly increased TBIL level, but a decreased ALB level. ALT (serum glutamic pyruvic transaminase) and AST (serum glutamic oxaloacetic transaminase) mainly appear in both liver and heart cells and are involved in liver disorders.^[22]^ ALT level marks the accumulation of liver fat, making the connection between ALT and liver fat being positive.^[23]^ Moreover, elevated ALT levels are referenced as being a marker for liver injury.^[24]^ Low AST level is associated with the relief of liver injury.^[25]^ It has been reported by Giannini *et al.* that the severity of cirrhosis is associated with the AST/ALT ratio.^[26]^ Bilirubin is also a major component of bile due to the breakdown of heme catabolism.^[27]^ Fatima *et al.* revealed that the cirrhosis patients had elevated TIBL levels,^[28]^ which is consistent with our results. ALB is used as a marker of nutritional status because of its association with the degree of malnutrition.^[29]^ More importantly, a decrease of ALB level was observed in the cirrhosis patients, which is in line with our results.^[30]^ Therefore, increases in ALT, AST, and TBIL levels as well as the decrease in ALB level play significant roles in cirrhosis, with the levels of both ALT and AST are associated with the severity of cirrhosis.

The present study also detected the elevated Tei index and plasma BNP levels, but low SV, EF, and E/A ratio in patients diagnosed with decompensated cirrhosis. Furthermore, the study observed that the Tei index was positively correlated with plasma BNP levels, and both Tei index and plasma BNP levels were positively correlated with Child–Pugh classification, while being negatively correlated with cardiac function and the prognosis in patients with decompensated cirrhosis. Cirrhosis has also been associated with the development of cardiac failure.^[4]^ SV, EF, and E/A ratio are typically applied for the assessment of ventricle function.^[31]^ In accordance with the findings, previous studies revealed that advanced cirrhosis patients showed reduced SV, EF, and E/A ratio.^[32,33]^ Furthermore, the Tei index is frequently applied to assess cardiac function.^[10]^ The Tei index consists of ICT and IRT divided by ET in each ventricle which is (ICT + IRT)/ET, showing that patients with cardiac dysfunction have elevated Tei index scores.^[11,34]^ Coinciding with the present study, Yanxin Su *et al.* observed that the patients dealing with uremia were accompanied with cardiac dysfunction, displaying both a longer ICT and IRT, but a shorter ET when compared with those of the control group.^[35]^ In addition, BNP levels revealed negative cardiac function in cirrhosis patients, and high BNP levels in patients with advanced cirrhosis.^[17]^ It was reported that the increase of BNP levels is parallel to the cirrhosis phase.^[36]^ Interestingly enough, a prior study conducted by Shi et al^[37]^ provided evidence that cirrhosis patients with both Child class B and C had elevated BNP levels when compared to that of Child class A, which has been consistent with the findings in the present study. To further the aforementioned information, both Tei index and plasma BNP levels have significant value for the evaluation of cardiac function in patients with decompensated cirrhosis. As cardiac dysfunction seems to potentiate the poor prognosis of cirrhotic patients, Tei index and plasma BNP levels might be useful for prognostic values in patients with decompensated cirrhosis.^[38]^ However, there are several limitations that were found, meaning there are still improvements remaining to be accomplished in the future. The omission of circulating markers of cardiac function (e.g., NT pro-BNP) resulted in a possible selection bias. Besides, using the short survival rate as outcome metric, which may be influenced by all variables, is a further limitation of the study. The results obtained should be examined in a larger sample size, and additional statistics methods and comprehensive analysis should be performed for more accurate results.

## 5 Conclusion

All in all, the study demonstrated that the Tei index combined with the plasma BNP levels was an effective assessment method in evaluating cardiac function in patients with decompensated cirrhosis, potentially shedding lights on the prognostic factor of decompensated cirrhosis, while enhancing the decompensated cirrhosis treatment.

## Acknowledgments

The authors want to show their appreciation to reviewers for their helpful comments.

## Author contributions

**Conceptualization:** Rui-Min Yang, Yi-Cheng Wang.

**Data curation:** Li-Kun Wang, Xiao-Fei An, Rui-Min Yang.

**Formal analysis:** Li-Kun Wang, Rui-Min Yang, Jie-Lin Yang, Yi-Cheng Wang.

**Funding acquisition:** Jie-Lin Yang.

**Investigation:** Li-Kun Wang, Su-Mei Zhang, Jie-Lin Yang.

**Methodology:** Li-Kun Wang, Xue-Liang Wu, Rui-Min Yang, Chao Han.

**Project administration:** Xue-Liang Wu, Su-Mei Zhang.

**Resources:** Xiao-Fei An, Xue-Liang Wu, Chao Han.

**Supervision:** Su-Mei Zhang, Chao Han, Jie-Lin Yang, Yi-Cheng Wang.

**Validation:** Xiao-Fei An, Rui-Min Yang, Jie-Lin Yang, Yi-Cheng Wang.

**Visualization:** Su-Mei Zhang.

**Writing – original draft:** Yi-Cheng Wang.

**Writing – review & editing:** Xiao-Fei An, Yi-Cheng Wang.
